# Mistletoe: From Basic Research to Clinical Outcomes in Cancer and Other Indications

**DOI:** 10.1155/2014/987527

**Published:** 2014-05-27

**Authors:** Matthias Kröz, Gunver Sophia Kienle, Gene Feder, Srini Kaveri, Steven Rosenzweig

**Affiliations:** ^1^Research Institute Havelhöhe (FIH), Havelhöhe Hospital, 14089 Berlin, Germany; ^2^Department of Oncology, Havelhöhe Hospital, 14089 Berlin, Germany; ^3^Institute for Social Medicine, Epidemiology and Institute for Social Medicine, Epidemiology and Health Economics, Charité University Medicine, 14089 Berlin, Germany; ^4^Institute of Integrative Medicine, University of Witten/Herdecke, 58313 Herdecke, Germany; ^5^Institute for Applied Epistemology and Medical Methodology, University of Witten/Herdecke, 79111 Freiburg, Germany; ^6^Academic Unit of Primary Health Care, School of Social and Community Medicine, Bristol BS8 2PS, UK; ^7^INSERM U1138, Université Paris Descartes, 75006 Paris, France; ^8^College of Medicine, Drexel University, Philadelphia, PA 19129, USA

The importance of integrative medicine in oncological care is increasing in accordance with growth of its evidence base. In central Europe, white-berried mistletoe (*Viscum album*) preparations not only are among the most common types of treatments used in integrative medicine but also have been among of the most commonly prescribed cancer treatments in Germany* per se* in 2010 [1]. By 2017, mistletoe preparations will have been used in the treatment of cancer patients for 100 years. Mistletoe is a historic, folk remedy, but the first recorded use in oncology was by the Dutch physician Ita Wegman who used a mistletoe extraction for the treatment of a breast cancer patient following a recommendation by Rudolf Steiner [2]. The constituents of the mistletoe berry include lectins, viscotoxins, glycoproteins, oligo- and polysaccharides, and membrane lipids [3]. The PubMed database alone lists more than 1,200 citations for “mistletoe,” with approximately 50 new entries each year. There are a multitude of laboratory-based studies demonstrating immune stimulation, cytotoxicity, proapoptotic effects, antiangiogenesis, and DNA stabilisation [3–7]; animal experiments have found tumor-reducing effects [8]. Recent observations of a potent anti-inflammatory effect of* Viscum album* via selective inhibition of COX-2 protein expression provide a further rationale for an antitumor role of mistletoe in view of the close relationship between cancer and inflammation [9]. More recent research focuses on new mistletoe extracts that contain lipophilic components, that is, triterpenes, shown to have strong cytotoxic effects in mouse models [10].

Recent years have seen growth in the number and quality of clinical research studies on mistletoe therapy reporting improved patient outcomes, including studies of its coadministration alongside chemotherapy to reduce adverse effects and to improve quality of life in breast cancer, ovarian cancer, and lung cancer patients [11, 12]. Its clinical efficacy regarding tumor control and survival has been contested [13]; other systematic reviews have been more positive, particularly with regard to health-related quality of life outcomes [14]. A 2013 randomised-controlled trial reported an increase in median survival time for patients with pancreatic cancer [15]. Further similar well-designed clinical trials on other cancer types are warranted.

This special issue covers a wide range of research from basic science to clinical outcomes for cancer and other indications. We intend it to provide a scientific forum to promote further research and publication in this field.

We have included three articles dealing with the important subject of safety and quantification of adverse events of mistletoe therapy. P. J. Mansky and colleagues present the results of a National Center for Complementary and Alternative Medicine phase 1 dose escalation study of combined whole mistletoe extract and gemcitabine treatment in cancer patients and interactions between these two agents. In a prospective observational study, M. L. Steele and colleagues report safety data on subcutaneous mistletoe therapy in a large cancer population. Another article of this group focuses on the important and as yet underreported issue of the safety of off-label intravenous* Viscum album* administration.

In a Serbian randomized-controlled study, W. Tröger and colleagues measured the impact of concomitant mistletoe treatment with adjuvant chemotherapy on health-related quality of life in breast cancer patients. In an Italian randomized controlled phase 2 study, A. Longhi and colleagues investigated the effect of* Viscum album* treatment compared with standard epirubicin chemotherapy on survival and health-related quality of life in patients with osteosarcoma.

Shifting upstream to laboratory research, R. Kuonen and colleagues present new* in vitro* findings on the effect of a* Viscum album* lipophilic extract containing triterpenes on fibroblast migration, which serves as a wound healing model. S. Baumgartner and colleagues studied the effects of a traditional (anthroposophical) pharmaceutical process involving high-speed mixing of mistletoe extracts; they used an* in vitro* model to discern tumoricidal from host resistance mechanisms of action.

We hope this special issue contributes to the evidence base of mistletoe therapy and stimulates further robust research to establish its place in oncological treatment.
